# Expanding access to substance use services and mental health care for people with HIV in Alabama, a technology readiness assessment using a mixed methods approach

**DOI:** 10.1186/s12913-022-08280-z

**Published:** 2022-07-15

**Authors:** Ellen F. Eaton, Kaylee Burgan, Greer McCollum, Sera Levy, James Willig, Michael J. Mugavero, Sushanth Reddy, Eric Wallace, Tom Creger, Stefan Baral, Susanne Fogger, Karen Cropsey

**Affiliations:** 1 Division of Infectious Diseases, Heersink School of Medicine, University of Alabama at Birmingham, BBRB 206-E | 845 19th Street South, Birmingham, AL 35205 USA; 2 Department of Surgery, Heersink School of Medicine, University of Alabama at Birmingham, 2000 6th Avenue South, Birmingham, AL 35233 USA; 3 Division of Nephrology, Heersink School of Medicine, University of Alabama at Birmingham, 1600 7th Ave S, Birmingham, AL 35233 USA; 4 Division of Infectious Disease Epidemiology, Department of Epidemiology, Bloomberg School of Public Health, John Hopkins University, E7146 | 615 N. Wolf Street, Baltimore, MD 21205 USA; 5 School of Nursing, University of Alabama at Birmingham, 1701 University Blvd, Birmingham, AL 35294 USA; 6 Department of Psychiatry, Heersink School of Medicine, University of Alabama at Birmingham, VH L107 | 1670 University Blvd, Birmingham, AL 35233 USA

**Keywords:** Mental health, Addiction, Telehealth, Healthcare delivery

## Abstract

**Background:**

Alabama is one of seven priority states for the National Ending the HIV Epidemic Initiative due to a large rural burden of disease. Mental health (MH) and substance use disorders (SUD) represent obstacles to HIV care in rural areas lacking Medicaid expansion and infrastructure. Evidence-informed technologies, such as telehealth, may enhance SUD and MH services but remain understudied in rural regions.

**Methods:**

We conducted a readiness assessment using a mixed methods approach to explore opportunities for enhanced SUD and MH screening using electronic patient reported outcomes (ePROs) and telehealth at five Ryan White HIV/AIDS Program-funded clinics in AL. Clinic providers and staff from each site (*N* = 16) completed the Organizational Readiness to Implement Change (ORIC) assessment and interviews regarding existing services and readiness to change. People with HIV from each site (PLH, *N* = 18) completed surveys on the acceptability and accessibility of technology for healthcare.

**Results:**

Surveys and interviews revealed that all clinics screen for depression annually by use of the Patient Health Questionnaire-9 (PHQ9). SUD screening is less frequent and unstandardized. Telehealth is available at all sites, with three of the five sites beginning services due to the COVID-19 pandemic; however, telehealth for MH and SUD services is not standardized across sites. Results demonstrate an overall readiness to adopt standardized screenings and expand telehealth services beyond HIV services at clinics. There were several concerns including Wi-Fi access, staff capacity, and patients’ technological literacy.

A sample of 18 people with HIV (PWH), ages 18 to 65 years, participated in surveys; all demonstrated adequate technology literacy. A majority had accessed telehealth and were not concerned about it being too complicated or limiting communication. There were some concerns around lack of in-person interaction and lack of a physical exam and high-quality care with telehealth.

**Conclusion:**

This study of PWH and the clinics that serve them reveals opportunities to expand SUD and MH services in rural regions using technology. Areas for improvement include implementing routine SUD screening, expanding telehealth while maintaining opportunities for in-person interaction, and using standardized ePROs that are completed by patients, in order to minimize stigma and bias.

## Background

Mental health (MH) and substance use disorders (SUD) in people with HIV (PWH) threaten HIV outcomes and stall efforts to end the HIV Epidemic, especially in rural states [1, 2]. Because untreated psychosocial comorbidities limit self-care, they impede individuals from attending to their HIV care needs. Alabama (AL) and six other states have been prioritized for the Department of Health and Human Services Ending the HIV Epidemic (EHE) initiative because of a substantial rural HIV burden. Most face additional challenges in treating psychosocial comorbidities due to a lack of public health infrastructure and healthcare providers, especially MH and addiction treatment providers. This treatment gap is due, at least in part, to rurality and lack of Medicaid expansion [3–6]. There is a well-established need for accessible programs that improve the diagnosis and management of MH and SUD in rural Americans. These programs would enhance individual and population health outcomes for communicable diseases like HIV, Hepatitis C, and other chronic diseases that disproportionately affect the rural U.S.

The UAB 1917 HIV Clinic implemented the routine capture of patient reported outcomes (PROs) at the point of care across several domains in 2007, such as depression (PHQ-9) and substance use (ASSIST) [7, 8]. PROs enable a more accurate diagnosis as compared to provider documentation [8]. The UAB 1917 HIV Clinic uses electronic PROs (ePROs) to identify and link PWH to MH and SUD services at the point of care, such as via screening, brief interventions, and referral to treatment (SBIRT) when high risk substance use is reported [9]. PROs enable standardized, high quality patient care and accurate reporting, which may be required by funders like Health Resources and Services Administration. Indeed, annual reporting on depression and substance use is required of Ryan White HIV/AIDS-funded HIV clinics. Yet screening for psychosocial comorbidities is just one of many barriers to care. Across Ryan White HIV/AIDS Program-funded clinics in Alabama, MH and SUD resources range from co-located specialty clinics, to referrals to clinics ≥ 30 miles away.

Telehealth (i.e., via smartphones, laptops, etc. to connect patients with providers outside of clinic visits) has been proven feasible and acceptable for delivering MH and SUD treatment, but is underutilized [10]. In rural and poor communities, telemedicine conducted via telephone (i.e., audio only) is the most accessible virtual option due to limited smartphone and computer access, as well as Wi-Fi capacity, which are essential for tele-video services [11, 12]. Hence, telephone only visits are essential for rural sites to continue to engage rural and aging patients.

To improve the care of MH and SUD in Alabama’s rural HIV clinics, we developed a multicomponent intervention called HIV ± Service delivery and Telemedicine through Effective PROs (+ STEP). The + STEP intervention will address multiple barriers to care by using a three-pronged approach: (1) standardized ePROs to more accurately screen for MH, via the PHQ-9 and generalized anxiety disorder (GAD-7) screeners, and SUD, via the ASSIST and alcohol use disorders (AUDIT-C) screeners; (2) training clinicians and staff on evidence-based care of MH and SUD; and (3) using telemedicine to expand service delivery when needed [13]. + STEP is intended to be integrated as the new standard of care at participating Ryan White HIV/AIDS Program-funded clinics.

The objective of this study is to assess whether key stakeholders, including patients and providers, are ready to implement + STEP at their sites and to determine the accessibility of and preferences for the intervention. Because many PWH in Alabama experience low literacy and income and are African American, data suggests they also experience greater barriers to healthcare information technology, like ePROs and Telehealth [14–16]. Indeed, structural racism serves as an obstacle to health information technology (IT) equity for much of the Deep South. The overall goal of this readiness assessment is to better understand the site’s current resources, protocols and procedures, and barriers to inform + STEP and ensure that it is appropriate for PWH in Alabama and the clinics that serve them. Findings may have broad implications for expanding evidence-based MH and SUD service delivery in other EHE prioritized states with a high prevalence of rural PWH.

## Methods

**Fig. 1:**
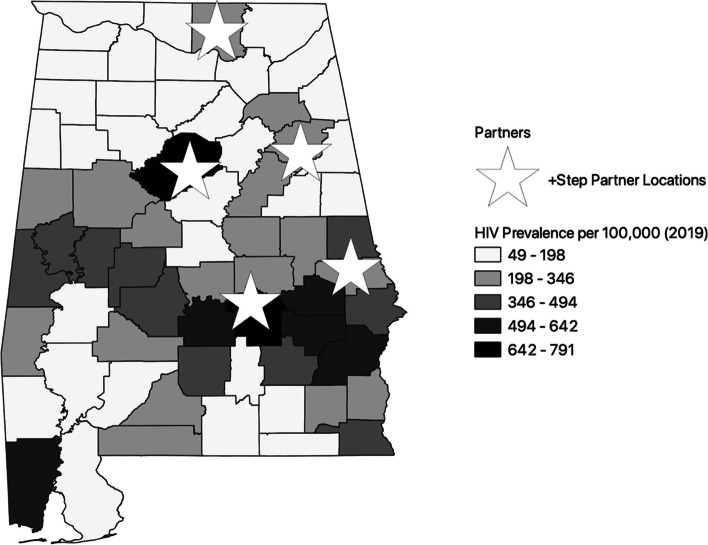
Map of HIV Prevalence in Alabama and participating Ryan White HIV/AIDS Program Clinics (Stars)*, This figure was developed by our study team by use of QGIS, an open source software

The current study was a prospective investigation using a mixed methods approach in order to evaluate key stakeholder’s readiness to implement + STEP at five HIV/AIDS clinics in Alabama. The + STEP intervention was offered to all nine clinics who receive Ryan White HIV/AIDS Program funds. Six sites expressed interest in participation, but upon the receipt of funding in September 2020 and following the outbreak of COVID-19, one of the six sites declined participation. This left five interested sites across the state: Health Services Center (Anniston), Unity Wellness Center (Opelika), Medical Advocacy and Outreach (Montgomery), Thrive Alabama (Huntsville) and UAB Family Medicine Clinic (Birmingham) (Table 1). The sites are located across the state, situated in areas of high HIV prevalence, as illustrated in Fig. 1, and have large catchment areas providing prevention and treatment services to persons in adjacent rural counties.

**Table 1:** Patients with HIV at Participating Sites in 2021

Summary of Sites Participating in + STEP	
Ryan White Funded Clinics in Alabama	PWH (n)
University of AL Family Clinic	300
Thrive Federally Qualified Health Services Center	956
Health Services Center	562
Medical Advocacy and Outreach	1819
Unity Wellness Center	428
TOTAL (Active PWH at Participating Sites)	4065

Data from 2020; 2021 data unavailable for this site

### Stakeholder interviews

Using convenience sampling, we recruited five key stakeholders at each clinic (*N* = 25), including clinicians, IT staff, and administrators, to participate in interviews and surveys about technological capacity and organizational readiness for change. Stakeholders were invited via email to participate in a virtual qualitative interview. The objective of the interviews was to query each clinic’s current resources and protocols, IT team structure, electronic medical records (EMR), data capture, Wi-Fi capabilities, existing MH and SUD screening procedures, and perceived barriers to telehealth. The virtual interviews lasted approximately 1 h. All interviews were guided by a semi-structured interview guide developed by the research team (supplemental) [17].

Following interviews, participants were asked to complete the Organizational Readiness to Implement Change (ORIC) assessment, a 12-item instrument based on Weiner’s theory of organizational readiness for change that is useful in determining a health organization’s readiness to adopt a new intervention. The instrument is assessment on a 5-point likert scale from Disagree (1) to Agree (5) and is helpful in determining employees’ perceptions on their organization’s ability to implement a new intervention. All interviews were audio recorded and transcribed for rapid qualitative analysis. Using five primary constructs from the Consolidated Framework for Implementation Research (CFIR), the study team relied on a deductive framework approach to analysis, wherein staff coded and summarized individual transcripts at each of the five constructs before synthesizing across all interviews. The five CFIR constructs used include: Compatibility, Available Resources, Design Quality & Packaging, Complexity, and Structural Characteristics [18]. Results were analyzed in an iterative fashion following each interview: After 3–4 interviews per site, the study team determined that there was no new information on clinic processes and protocols that would assist in determining readiness. Hence, recruitment was completed at that time. Survey data were collected via an anonymous link using Qualtrics Software. Participants responded anonymously but provided the name of their organization so that we could link results with a clinic’s readiness to adopt + STEP.

### Patient Surveys

We recruited five patients receiving care from each participating site (*N* = 25) to complete an anonymous survey on acceptability and accessibility of ePROs and telehealth services via Qualtrics Software. Key stakeholders at clinics identified PWH who have personal experience with MH or SUD and referred them to the study team for recruitment. The study team contacted patients via telephone or email, based on patient’s recorded contact preferences, and were asked to participate in an approximately ten minute survey. The survey covered a range of questions around technology access, comfort, health literacy, and financial barriers. We used the Telemedicine Comfort Assessment adapted from Irfan et al., a survey that uses a Likert scale to assess three constructs: communication, device maintenance, and the performance of complex tasks with a technological device, such as a smart phone [19]. The Telemedicine Comfort Assessment also includes a Brief Health Literacy Assessment (supplemental) [20] and has been validated in patients with cancer living in Alabama. We also adapted questions from Gurupur et al. to address the likeability of telemedicine services (supplemental) [21]. Following discussion with key stakeholders, we added additional questions in order to understand difficulties with dropped calls, Wi-Fi outages, and lapses related to financial constraints. We asked about telehealth decision making as it relates to the COVID-19 pandemic, competing demands (e.g., work, gas), and experience with telehealth during the pandemic.

All surveys administered in this study are not under licence and were readily accessible at the sources referenced in text.

This study’s protocols, documents, and forms used were approved by the UAB Institutional Review Board.

## Results

### Stakeholder results

A total of 16 stakeholders participated in in-depth interviews about their organizations’ existing screening and treatment protocols and organizational readiness to adopt a new standard of care. While we originally set a goal to interview 25 key stakeholders (five per site), the study team obtained no new information on clinic protocol and procedures after three to four interviews per site. A summary of existing PROs, telehealth infrastructure, and perceived barriers to + STEP are summarized in Tables 2, 3, and 4. All clinics reported using a mixture of paper and electronic systems (e.g., electronic health record) to capture PROs (Table 2), and there was some interest in the use of tablets to reduce double entry and data entry error. We found that all clinics use the Patient Health Questionnaire, either the 2 (PHQ2) or 9-item (PHQ9), to screen all PWH for depression at least annually. However, screening for substance use and other MH disorders beyond depression was less standardized and less frequent (Table 2) [22]. Stakeholders indicated that only two of the five sites utilized telehealth services prior to the COVID-19 pandemic; the remaining three sites began adopting telehealth as a result of COVID-19 and continue to use it as needed (Table 3). All five sites use telehealth for medical care, and MH and SUD telemedicine services are offered at most (*N* = 4 sites). However, participating staff and clinicians had common concerns about patient technical literacy (*N* = 3 participants), Wi-Fi capacity (*N* = 2), and staffing requirements (*N* = 2).

**Table 2:** Universal Substance Use and Mental Health Validated Screening Tools employed by Participating Clinics

Site	Data Capture Method	Existing Screening Tools for ALL RW Patients	Frequency
1	EMR	Depression: PHQ-2	Annually
		Anxiety: GAD-7	Annually
		Alcohol: N/A	–
		Substances: N/A	–
2	Paper and EMR	Depression: PHQ-9	Annually
		Anxiety: GAD-7	Annually
		Alcohol: N/A	–
		Substances: N/A	–
3	Paper and EMR	Depression: PHQ-9	Annually
		Anxiety: N/A	–
		Alcohol: UNCOPE	Annually
		Substances: UNCOPE	Annually
4	EMR	Depression: PHQ-9	Annually
		Anxiety: N/A	–
		Alcohol: N/A	–
		Substances: N/A	–
5	Paper and EMR	Depression: PHQ-2	Annually
		Anxiety: N/A	–
		Alcohol: N/A	–
		Substances: N/A	–

Surveys were developed on site and have not been validated

**Table 3:** Existing Telemedicine Services at Participating Ryan White HIV/AIDS Program (RWHAP) Sites

RWHAP Site	Telehealth Existed prior to COVID	Initiated Telehealth during COVID	Audiovisual Telehealth	Telehealth MH Services	Telehealth SUD Services
1	x	–	x	x	–
2	–	x	x	x	–
3	–	x	–	–	–
4	–	x	x	x	–
5	x	–	x	x	–

**Table 4:** Existing Infrastructure and Perceived Barriers to ePRO and Telehealth Services for 5 Ryan White HIV Clinics in Alabama

Clinic	IT Team	EMR	Data Capture	Central Storage	Secure WI-FI	PRO Administration	Use of tablets	Quality assurance process	Perceived Barriers
1	1 manager 2 IT desktop 1 admin	Intergy Greenway Health	EMR	Yes	Yes	Staff administered	No	Yes, Monthly	Patient technical literacy
2	1 staff	CareWare, iConnect	Paper and EMR	Yes	Yes	Patient administered	No	Yes, Quarterly	Wi-Fi capacity Tablet security COVID precautions
3	1 staff	CERNER CareWare	Paper and EMR	No	Yes	Staff administered	No	Yes, As Needed	Staffing requirements Patient technical literacy Health system bureaucracy
4	1 staff, 1 contractor	eClinical Works	EMR	Yes	Yes	Staff administered	No	Yes, Annually	WI-FI capacity Data entry Staffing Requirements COVID precautions

One site uses tablets for research only and another discontinued the use of tablets due to patients’ technical challenges

According to the ORIC assessment results, the majority of respondents report that their respective clinic is ready to adopt + STEP as the new standard of care. Most participants agreed or somewhat agreed with all statements (see supplement), indicating general receptiveness to change, commitment to implementing the program, and confidence in their ability to implement the program effectively. Most respondents (75%) agreed with the statement that “People who work here feel confident that the organization can get people invested in implementing + STEP.” Only one respondent (6%) indicated disagreement with the statement that “People who work here feel confident that they can manage the politics of implementing + STEP,” while all other respondents (94%) either agreed or somewhat agreed.

### Patient results

A total of 18 PWH participated in the surveys: most were ages 22 to 44 years (50%) and over 45 years old (44%). A majority (61%) identified as male, and 61% identified as African American. Almost all reported using telehealth in the past (89%, *N* = 16). Of those who have utilized telehealth services in the past, 50% (*n* = 8) have used telephone conferencing, 19% (*n* = 3) have used video conferencing, and 31% (*n* = 5) have used both. Of the respondents who had participated in a video conferencing health encounter (*n* = 8), 88% (*N* = 7) report using a mobile phone for this service. The majority of all respondents who answered the question (82%, *n* = 13) report being comfortable using some method of telehealth; however, many of these respondents still reported concerns (Table 5). Across participants, the top concerns about using telehealth include lack of a physical exam (75%, *n* = 9), fear of low quality of care (67%, *n* = 8), and missing in-person interaction (58%, *n* = 7) (Table 5).

**Table 5:** Concerns about Telehealth

Question	Not Very Important		Somewhat Important		Very Important	
Missing in-person interaction	0%	0	42%	5	58%	7
Lack of physical examination	0%	0	25%	3	75%	9
Potential for technical issues	25%	3	50%	6	25%	3
Fear of low quality of care	17%	2	17%	2	67%	8
Discomfort with technology	42%	5	33%	4	25%	3
Security/privacy concerns	42%	5	33%	4	25%	3
Other	100%	1	0%	0	0%	0

In terms of telehealth decision making, a majority of patients disagreed with the i dea that telehealth was too complicated (78%, *n* = 14), and disagreed that money saved by not leaving work would affect their telehealth decisions (67%, *n* = 12) (Table 6). Responses were mixed, though, on whether money saved on gasoline would affect their decision to use telehealth. Many participants disagreed with concerns about being able to understand the doctor using telehealth (83%, *n* = 15) and about the doctor being able to understand them (83%, *n* = 15). Responses were highly variable regarding the statements that “Concerns about COVID-19 would affect my decision to use telehealth” and that “I would prefer to see a physician sooner through telehealth than wait to see a physician in-person” (Table 6).

**Table 6:** Telehealth Concerns and Decision Making

Question	Disagree		Somewhat Disagree		Neutral		Somewhat Agree		Agree	
The money saved in time away from work would affect my decision to use telehealth	67%	12	0%	0	6%	1	11%	2	17%	3
The money saved on gasoline would affect my decision to use telehealth	44%	8	11%	2	0%	0	11%	2	33%	6
Concerns about the COVID-19 pandemic would affect my decision to use telehealth	28%	5	11%	2	11%	2	17%	3	33%	6
I would prefer to see a physician sooner through telehealth than wait to see a physician in-person	22%	4	28%	5	6%	1	11%	2	33%	6
I am concerned about being able to understand what the doctor says through telehealth	50%	9	33%	6	6%	1	0%	0	11%	2
I am concerned about the doctor being able to understand me through telehealth	44%	8	39%	7	6%	1	0%	0	11%	2
The idea of telehealth sounds too complicated	61%	11	17%	3	0%	0	17%	3	6%	1

In terms of technology use, most participants were comfortable using a cell phone for texting (83%, *n* = 15), email (83%, *n* = 15), and password management (89%, *n* = 16). The majority were also comfortable installing new apps (78%, *n* = 14), making tele-video based calls with their phone (67%, *n* = 12), and connecting to a free Wi-Fi network (61%, *n* = 11) (Table 7).

**Table 7:** Comfort with Technology

Question	Very Uncomfortable		Uncomfortable		Neutral		Comfortable		Very Comfortable	
Using a cell phone to send a text message	11%	2	0%	0	6%	1	6%	1	78%	14
Checking email on my computer	11%	2	0%	0	6%	1	28%	5	56%	10
Checking email on my phone	11%	2	0%	0	0%	0	22%	4	67%	12
Installing an app on your phone or computer	11%	2	11%	2	0%	0	6%	1	72%	13
Commenting on a friend's post on social media	11%	2	11%	2	6%	1	11%	2	61%	11
Connecting to a free Wi-Fi network	11%	2	17%	3	11%	2	17%	3	44%	8
Making a video-based call with your phone	17%	3	11%	2	6%	1	17%	3	50%	9
Changing the password to your phone or other electronic device	6%	1	6%	1	0%	0	28%	5	61%	11

## Discussion

Our findings demonstrate that, across Alabama, PWH, and the Ryan White HIV/AIDS Program-funded clinics who serve them, are generally willing and able to implement technology-enhanced, evidence-based MH and SUD screening and telehealth services. All five participating HIV clinics in AL reported organizational readiness to adopt + STEP, which includes ePROs; targeted MH and SUD trainings for clinicians and staff; and telehealth services to expand access to care. Further, these mostly rural PWH reported an acceptable level of comfort with technology and telehealth interactions, including virtual health-related communications with providers. Our results suggest that clinics serving rural populations can and should consider integrating technology into routine patient care to improve the diagnosis and treatment of underdiagnosed and treated MH and SUD. Specifically, advancing access to care for underlying mental illness is an important next step in eliminating the rural mental health and drug use crises, achieving individual HIV viral load outcomes, and preventing HIV transmission, which are all critical for ending the HIV epidemic.

Staff and clinicians highlighted key considerations within their existing clinic capacity and workflow that will impact technology-based interventions in both participating clinics (Table 1) and rural areas, more broadly. First, there is significant diversity in the type and number of IT support staff at participating clinics, ranging from one IT generalist to four dedicated IT staff. This difference in IT capacity will influence the implementation and sustainability of ePROs, collected via tablet or desktop device, and on- site telehealth support. Second, some sites use multiple electronic databases to collect and maintain clinical documentation and reports; rural clinics should anticipate these fragmented systems when incorporating any new survey into care [23]. Adding another layer of complexity, many clinics collect data (e.g., sociodemographic and screening forms) both on paper and electronically. This is done to accommodate patients and staff who prefer paper and to account for technology challenges, like system outages. This hybrid approach to documentation adds another competing demand for limited staff in terms of storing, scanning, and/or manually entering paper forms into EMRs. But hybrid data entry may be the most appropriate accommodation in some rural clinics where real-time entry is not feasible due to limited technology and/or staff support [24]. Lastly, although all clinics conduct some form of MH and/or SUD screening, most clinics’ protocols involve a staff member reading the MH and SUD screening questions to patients. Ideally, PROs are self-administered and self-reported in order to reduce the social desirability and response bias and diminish feelings of stigma and shame around mental illness and substance use [8, 25]. Patient administered surveys are not the standard at most sites, leaving room for improving accuracy of screening services.

When focusing more specifically on + STEP, most stakeholders felt confident in their clinic’s ability to implement the strategy. Yet, there were several concerns that deserve additional consideration including interruptions in Wi-Fi capacity, both in clinics and patients’ homes, and patients’ technology literacy. There were also clinic-level concerns about staffing requirements and staff burden. It is notable that this study was conducted early in the course of the COVID-19 pandemic when many clinics and businesses faced devastating staffing shortages due to staff sickness, burnout, and early retirement. One of the greatest challenges to rural telehealth for mental health nationwide is a lack of trained specialists, a concern voiced by many of our stakeholders [26]. Unfortunately, these issues remain almost two years into the COVID-19 pandemic, making them important considerations for any clinic adding innovations.

Despite stakeholder concerns about technology literacy, patients expressed overall comfort with technology and telehealth. Due to clinic advances during the COVID-19 pandemic, the majority of patients in the study had participated in a telehealth visit previously, with the majority of them using a telephone for an audio-only encounter. Most patients felt comfortable using many devices, including smartphones, and performing several tasks on such devices. This is in contrast to concerns that technology widens the digital divide for African Americans: African Americans are less likely to use technology for health information, such as patient portals [27]. Often, access to a personal computer and internet access are the greatest barriers to engaging in health-related technology so comfort with a device is not equivalent to access [28, 29], especially for patients living in poverty. Although many findings were encouraging, it is important to note that many patients expressed telehealth concerns: missing out on the physical exam, having limited in-person interaction, and receiving lower quality healthcare. These concerns should be addressed with patients individually before providing telehealth services.

Oftentimes, specialty care is not accessible in rural areas, making + STEP an important innovation in terms of integrating services into routine HIV care [26]. Integrated care eliminates the false dichotomy between physical and mental health (e.g., body and mind). Integrated care can also improve mental health service access and utilization because people seek primary care (e.g., HIV care) more often than mental health or addiction specialty services. There are also more rural primary care sites, including HIV clinics with unique federal funding, than there are rural mental health clinics. Capitalizing on this integration of screening and treatment under the umbrella of a Ryan White HIV/AIDS Program-funded clinic has the potential to reach more patients and provide more accessible care than referrals to costly, out-of-town specialty clinics.

Further, telehealth can extend access to integrated services. To equip clinics for telehealth, clinic leadership should offer trainings to patients and staff in the use of telehealth and relevant applications and provide on-site devices for patients to participate in telehealth with off-site specialists, such as a psychiatrist who provides virtual services from an urban clinic. Clinics can alleviate concerns about limited interaction and physical exams by developing hybrid services: offering in-person visits for initial and annual visits and telehealth services for interval and as needed assessments. Lastly, stakeholders and policy makers must continue to support audio-only encounters through policy and fair compensation. This is imperative to prevent a deeper digital divide for rural Americans and racial/ethnic minorities for whom smartphones and Wi-Fi are inaccessible [12]. As successive spikes and surges in the COVID-19 pandemic threaten clinic closure and staff shortages, the use of telephones will allow patients of all races, income levels, and technology comfort levels to continue to access healthcare. This is true for both public health emergencies and personal challenges (e.g., lack of transportation).

Our study included a number of limitations. Foremost, we initiated the study in September of 2020, relatively soon after the onset of the COVID-19 pandemic, a time of increased stress and anxiety for healthcare providers across the globe. One participating clinic declined further involvement at least in part due to the pandemic, leaving a smaller sample—five participating clinics. Additionally, three of the five clinics adopted telehealth technologies during the pandemic, which we planned to initiate as part of + STEP. The recent adoption of such innovations could have skewed the responses of participating stakeholders. Further, as telehealth is scaled up and COVID-19 becomes less of a risk to in-person patient care, clinics may experience new or more complex barriers that were not reported early in adoption. Because many clinics include few staff, there were few eligible stakeholders at participating clinics, and a convenience sample was used for both patient and stakeholder assessments. Clinic leadership from each site identified stakeholders including a mental health provider, IT staff, administrator, and clinician; mental health providers identified eligible patients for participation. This convenience sample could limit the generalizability of these findings.

## Conclusions

The U.S. is facing a syndemic of disease that weighs most heavily on rural and poor communities: mental health and drug use crises, a recalcitrant HIV epidemic, and COVID-19. Health technology has the potential to extend evidence-based care of mental illness and addiction into underserved areas and can overcome barriers imposed by geography and public health emergencies. In general, HIV clinics serving large numbers of rural persons in Alabama are able to scale up technology for mental health and substance use services, and PWH report overall comfort with telemedicine and related devices. Stakeholders should anticipate several considerations when integrating health technologies in rural clinics: fragmented EMRs, hybrid documentation (paper and electronic), Wi-Fi limitations, patient preferences for human touch, and policy changes that may limit or extend COVID-era provisions for telehealth usage and compensation. Anticipating these challenges and proactively seeking solutions might allow for more effective implementation of programs akin to + STEP in other EHE prioritized states with high rural HIV prevalence, allowing for improved adoption, uptake, and resulting MH, SUD, and HIV outcomes.

## Data Availability

All data is stored in a secure location. Data may be shared, without patient identifiers, contingent upon approval by the UAB Institutional Review Board. For inquiries, please contact the corresponding author.

## Abbreviations

EMR: Electronic Medical Record
EHE: Ending the HIV Epidemic
IT: Information Technology
PROs: Patient Reported Outcomes
ePROs: Electronic Patient Reported Outcomes
PWH: People with HIV
AL: Alabama
SUD: Substance Use Disorders
MH: Mental Health Disorders
PHQ9: Patient Health Questionnaire-9
ORIC: Organizational Readiness to Implement Change
UABI: University of Alabama at Birmingham
VL: Viral Load
ASSIST: The Alcohol Smoking and Substance Involvement Screening Test
AUDIT-C: Alcohol Use Disorders – Condensed
SBIRT: Screening, Brief Interventions, and Referral to Treatment
+ STEP: HIV Service delivery and Telemedicine through Effective PROs
CFIR: Consolidated Framework for Implementation Research
GAD7: Generalized Anxiety Disorder-7
UNCOPE: Used Neglected Cut Down Objected Preoccupied Emotional Discomfort
